# Wins and losses in intergroup conflicts reflect energy balance in red-tailed monkeys

**DOI:** 10.1098/rstb.2021.0152

**Published:** 2022-05-23

**Authors:** Michelle Brown, Ronnie Steinitz, Melissa Emery Thompson

**Affiliations:** ^1^ Department of Anthropology, University of California, Santa Barbara, 552 University Rd, Santa Barbara, CA 93106-3210, USA; ^2^ Department for the Ecology of Animal Societies, Max Planck Institute of Animal Behavior, Bücklestraße 5, 78467 Konstanz, Germany; ^3^ Department of Anthropology, University of New Mexico, MSC01‐1040, 500 University Blvd NE, Albuquerque, NM 87131, USA

**Keywords:** between-group competition, energetics, evolutionary game theory, resource defense

## Abstract

The energetic costs and benefits of intergroup conflicts over feeding sites are widely hypothesized to be significant, but rarely quantified. In this study, we use short-term measures of energy gain and expenditure to test whether winning an intergroup encounter is associated with greater benefits, and losing with greater costs. We also test an alternative perspective, where groups fight for access to large food sources that are neither depletable nor consistently monopolizable: in this case, a group that has already fed on the resource and is willing to leave first (the loser) is supplanted by a newly arrived group (the winner). We evaluate energy balance and travel distance during and after encounters for six groups of red-tailed monkeys in Kibale National Park, Uganda. We find that winning groups experience substantial energetic benefits, but do so to recoup from earlier deficits. Losing groups, contrary to predictions, experience minimal energetic costs. Winners and losers are predictable based upon their use of the contested resource immediately before the encounter. The short-term payoffs associated with these stressful conflicts compensate for any associated costs and support the perception that between-group contests are an important feature of social life for species that engage in non-lethal conflicts.

This article is part of the theme issue ‘Intergroup conflict across taxa’.

## 1. Introduction

When intergroup conflicts are severe, the costs of participation are clear: individuals risk death or wounding (e.g. chimpanzees [1], wolves [2], ants [3], banded mongooses [4]). For other species, these conflicts are non-lethal but nonetheless can dramatically alter the fitness of group members through changes in access to territory, den or nesting sites, mates, or other fitness-limiting resources [5–7]. These examples, however, come from just a small fraction of the species that routinely engage in intergroup contests [8]. For most species, the question remains: what are the costs and benefits of winning or losing such a contest?

Evolutionary game theory is useful for predicting which opponent wins or loses a contest [9–11]. It proposes that most animal conflicts are settled by asymmetries in resource-holding potential, expected payoffs or both factors. More specifically, the stronger and/or more motivated contestant holds its ground and may attack if necessary, but the conflict ends when the weaker and/or less motivated contestant retreats [12]. Whereas both contestants may experience costs associated with agonism (e.g. energy expended in chasing or missed feeding opportunities), it is generally assumed that only winners experience benefits (such as access to food, shelter or mates) [10]. This perspective stems partly from the way in which a ‘win’ is defined: after the separation of the two groups, the winner is the contestant that remains in the encounter location with continued access to local resources, while the loser is the group that departs first [8,13–15]. If the groups depart simultaneously, the winner is the group that continues moving in its pre-contest direction, while the loser moves away at a larger turning angle. In both cases, the underlying assumption is that losers experience costs, but no benefits.

There is at least one context, however, in which the loser of an intergroup contest reaps the same benefits as the winner: when conflicts occur over a resource that neither contestant can deplete or monopolize for long periods. For instance, bands of feral horses compete for access to water holes during the dry season and due to a ‘respect for ownership’ convention, whichever group arrives first can successfully monopolize the resource until all adult members have satiated themselves—even if the first group is larger or smaller than the challenging group [16]. In this case, both groups are able to drink their fill, though the recently arrived group is forced to wait until the departure of the first group. Crucially, the first group to depart (which is typically scored as the losing group) does so because it is satiated. This pattern upends the assumption that losing groups reap no benefits, and demonstrates the importance of documenting the true costs and benefits of winning and losing.

Among group-living primates, conflicts over resources that are not immediately depletable or permanently monopolizable may be more common than is generally assumed. Primate groups often fight with neighbours for access to specific food trees [17] or general feeding areas [6,18]. Food-related conflicts are most likely to occur when groups feed predominantly from trees that are widely dispersed [19] and are the last large crops available in the home range [17,20,21]. In particular, groups fight for access to resources that are large enough to feed most or all group members [22,23]. Large trees produce fruit, flowers, or leaves for several consecutive weeks and are not completely depleted after a foraging bout by a single group [24]. Moreover, because most primates travel hundreds or thousands of metres each day to feeding patches of different plant species in order to meet their nutritional goals [25], groups are unlikely to be able to monopolize a single resource for an entire day or fruiting period. As a result, these resources may engender intergroup contests in which both winners and losers reap foraging benefits. Unlike the example of the watering hole contested by feral horse bands [16], to the best of our knowledge, the availability of contested foods has never been measured both before and after a primate intergroup encounter. As long as the losing group has already foraged at the encounter location, its departure may reflect the reduced value of the resource [24] – i.e. a reduced motivation to monopolize access to a feeding patch in which they have eaten their fill—rather than subordinate status or lower resource-holding potential than the newly arrived, winning group.

To test this proposal, we evaluate the travel patterns and energy balance of red-tailed monkey (*Cercopithecus ascanius*) groups both before and after intergroup conflicts, which focus on access to feeding sites [17] and thus should affect patterns of energy gain. We evaluate levels of urinary C-peptide, which is a by-product of insulin production that rises in response to post-prandial glucose surges and indicates energy balance, which is the difference between energetic inputs and outputs [26,27]. Animals with better access to food—as would be expected for a group that wins a conflict over access to a feeding site—have higher C-peptide levels: baseline levels are higher in individuals that are gaining weight [28], when animals have greater access to carbohydrate-rich food sources [29–31] and when feeding competition is weak [32]. Though C-peptide indicates rapid changes in energy gain, it is also important to explore changes in energy expenditure. Using indirect measures, a previous study found that losing groups travelled further on the day of the conflict [8], but it is unclear whether they experience energy shortfalls significant enough to cause them to increase their search for food on the subsequent day.

From the traditional perspective of contest outcomes, both groups may experience energetic costs of engaging in a conflict—such as energy lost through chasing or fighting, and missed opportunities for feeding—but only the winning group reaps energetic benefits (hereafter referred to as the ‘winners benefit’ perspective) [10]. If correct, then winning groups will have higher C-peptide levels after an encounter than losing groups (P1a), or will experience greater gains in C-peptide levels than losing groups (P1b), because access to the contested resource should at least compensate for energy lost during the encounter. Losing groups should experience a decline in their C-peptide levels (P1c) because they are prevented from accessing the contested resource. If the costs of losing are substantial, then losing groups should travel further the next day to make up for the energy expended and lost foraging time during the conflict (P1d).

In contrast, our alternative perspective predicts that both groups experience feeding benefits and thus there should be no difference in their post-conflict C-peptide levels (P2a; hereafter referred to as the ‘everybody benefits’ perspective). The only predictable difference between winners and losers (aside from post-conflict movements) should be pre-conflict movements. I.e., if defended feeding sites are indeed non-depletable and non-monopolizable over long periods, and losing groups leave because they have already fed upon the resource (not because they are subordinate, as in [16]), we predict that groups will travel farther in the 30 min before encounters that they win compared to encounters that they lose (P2b). We also predict that their travel distance the next day will be unaffected by wins and losses (P2c).

Many intergroup conflicts end in a draw, rather than a win/loss, where both groups leave simultaneously and there is no difference in their turning angles (either both turn around or both move forward), and neither group gains access to a contested resource [33]. Evolutionary game theory is silent on this issue, but previous analyses find that draw outcomes are more likely to occur when the contestants are equally matched in resource-holding potential and expected payoffs. We predict that encounters ending in draw outcomes are marked by the absence of energetic benefits, and thus both groups experience only costs associated with the conflict and a reduction in C-peptide levels (P3a). As a result, both groups should travel greater distances on the following day to compensate for the costs of the encounter (P3b).

## 2. Methods

### (a) Study site and species

We conducted this study at the Ngogo research station (0°29′ N 30°25′ E) in Kibale National Park in western Uganda. Ngogo consists largely of old-growth rainforest intermixed with small patches of regenerating woodlands and riparian swamps [34]. It experiences two wet and two dry seasons each year.

Red-tailed monkeys are small-bodied (females: 2.7 kg, males: 3.8 kg), arboreal monkeys commonly found in both regenerating and old-growth forests in central and eastern Africa [35]. Groups typically contain one adult male, several adult females, subadults and immatures (electronic supplementary material, table S1; [36]). Females are philopatric but unlike other cercopithecine primates [37], agonistic interactions occur too rarely to permit identification of dominance ranks in this population (M. Brown, personal observation). They primarily consume fruit and insects with smaller quantities of other plant parts. Ngogo is characterized by a high density of red-tailed monkeys (5.27 groups/km^2^, mean 16.4 adults and subadults per group) and groups occupy relatively small home ranges (95% autocorrelated kernel density estimate, calculated using CTMMweb [38]: mean 0.35 ± s.d. 0.06 km^2^, *N* = 6 groups) with ∼26% overlap between any two neighbouring groups [17]. Though groups at Ngogo encounter a neighbour approximately every 1.5 days, only 42% of encounters escalate from vocal threats to aggressive chases, which occur at high-intensity feeding sites (i.e. locations where a large fraction of the group's monthly feeding records occur; [17,39]). Adults, subadults and juveniles of both sexes participate in aggressive encounters. There do not appear to be stable dominance relationships between groups (M. Brown, personal observation).

### (b) Data collection

We collected the data for this analysis as part of a broader study on intergroup conflict in six neighbouring groups of red-tailed monkeys from January 2012 through June 2015. All mature animals are individually recognized using the shape of the white nose spot, nipple characteristics (for females) and scars or other injuries. We followed 2–3 groups simultaneously for one to two weeks each month for multi-month periods (mean 4.9 ± s.d. 1.1 months, *N* = 7 periods), with 11–17 months between successive periods for a group. On every follow day, 2–4 observers tracked each group from dawn until dusk and noted the presence or absence of each individual, as well as whether each female was carrying and nursing an infant. We recorded the location of the group every 30 min as the point around which the majority of group members were clustered; we determined location using a 50 × 50 m gridded map of the trail system and by pacing to the nearest trails, or by using a hand-held GPS unit and later converting the UTM coordinates to the grid cell format. We recorded all foraging activity by group members during a 5 min window every half hour, including the plant species and part eaten.

Observers recorded details of intergroup encounters, which we define as periods when the edges of two groups are ≤50 m apart, using a pre-printed template to ensure consistency across observers [17]. These details include the start and end times, the identity of the opposing group (if known), whether any chases or physical contact occurred between groups, and the location of the encounter. Observers spaced themselves out along the leading edge of the focal group as well as behind this edge in order to maximize our ability to track the events of these sometimes chaotic encounters. Encounters typically last (median) 53 min (inter-quartile range: 31–82 min) [39]. We also followed the opposing group for 60 min after the end of the encounter to track its movements.

We collected fresh urine samples from mature individuals opportunistically throughout the day by pipetting droplets from low-lying vegetation immediately after excretion. We stored the samples on ice until 1700 h, at which time they were transferred to a −12°C freezer at the camp site. MB transported samples 1–2 times per year to the Hominoid Reproductive Ecology Laboratory at the University of New Mexico and we measured C-peptide levels with commercial radioimmunoassay kits (Millipore Sigma, Burlington, MA, USA) using the manufacturer's instructions. Most samples fell near 50% binding on the standard curve at a 1 : 5 dilution in assay buffer, but some samples required varying dilutions from 1:1 to 1:100. The inter-assay CV of the high and low quality controls was 4.6% and 8.6% (*N* = 44 assays) and the mean intra-assay CV of samples was 2.3 ± 2.2% (*N* = 1138 samples). Assay sensitivity was 0.065 ng ml^−1^ and no samples fell below this threshold. We verified parallelism by comparing binding from serial dilution of a sample (*y* = −47.4*x* + 183.8) to the standard curve (*y* = −45.9*x* + 179.8, *t* = 0.43, d.f. = 8, *p* = 0.68). Assay accuracy, as determined by the recovery of a sample added in duplicate to all points of the standard curve, was 101.8 ± 1.5% (mean ± s.d., *N* = 6). We measured the specific gravity of each urine sample using a handheld refractometer (Atago USA Inc., Bellevue, WA, USA) and corrected C-peptide values for water content as follows: C-peptide x [(SG_population_ – 1)/(SG_sample_ – 1)] [40], where SG_population_ is the mean specific gravity per group in a multi-month period.

### (c) Data analysis

Many factors are likely to affect whether a group wins or loses an intergroup encounter. In blue monkeys (*Cercopithecus mitis*), which are closely related to red-tailed monkeys, groups tend to win conflicts when they are larger than the opposing group and when the encounter occurs closer to the centre of the home range [33]. In other species, groups win when more individuals participate aggressively [41], when resident males are larger [14], or when groups are more strongly limited by food availability [21]. While all of these are important considerations, in this analysis we focus on the roles of group-wide energetic condition and immediate use of the encounter location (indicated by whether the group was stationary or travelling before the encounter), as these are novel predictors that have not previously been examined.

We defined four types of intergroup encounter outcome based upon the movements of the groups, relative to each other and/or to their pre-encounter travel direction [13,14,18]. ‘Displacements’ occurred when one group (the winner) stayed in the encounter location for at least 30 min after the departure of the other, losing, group (*N* = 38 encounters). ‘Deflections’ occurred when both groups moved away after the encounter (*N* = 7), but the winner continued moving within 45° of its original travel direction while the loser turned around to retreat into its home range. ‘Mutual avoid’ outcomes occurred when both groups turned around and retreated (*N* = 38), and ‘mutual ignore’ occurred when both groups continued moving forward (*N* = 1). Both displacements and deflections are considered ‘decided’ outcomes, while mutual avoid and ignore are ‘draw’ outcomes.

We sought to test whether urinary C-peptide levels change after an intergroup encounter, so we needed to determine the appropriate window of time represented by a urine sample. In humans, C-peptide spikes approximately 2 h after ingesting carbohydrates [27] but it was unclear whether this window would be appropriate for wild monkeys. Red-tailed monkeys have a 20–39 h digestive passage time [42], which means that complex carbohydrates digested in the large intestine could produce insulin surges a full day after a food item is consumed. At the other end of the temporal spectrum, cercopithecine monkeys produce high levels of amylase in their cheek pouches, allowing for rapid starch digestion less than 10 min after consumption [43]. Our preliminary investigation of the temporal lag between feeding behaviour and C-peptide levels indicates that this hormone responds to foods consumed both on the day of excretion as well as the previous day (M Brown, R Steinitz 2021, unpublished analyses). Consequently, we chose to track C-peptide levels both on the day of the encounter as well as the following day, in order to test whether encounter outcomes correspond to energy balance.

For each urine sample, we determined whether an intergroup encounter occurred on the same or following day; for those samples with an associated encounter, we calculated the difference between the time at which the sample was voided and the start of the encounter. We assigned negative and positive values for ‘time to encounter’ for samples produced before and after an encounter, respectively. We did not have enough urine samples from both interacting groups per encounter to compare their changes in C-peptide levels, so our analyses investigate general changes experienced by groups on days when they won, lost, or experienced a draw outcome. We also included samples from control periods: these were 2-day periods throughout the study during which we had collected multiple urine samples on each day, and when there was no intergroup encounter. The control samples allowed us to determine the normal patterning of C-peptide responses and served as a useful comparison with the intergroup encounter-associated samples. To facilitate this comparison, we assigned a fake intergroup encounter number to each 2-day control period and designated the fake encounter start time as noon on the first day. This was a reasonable approximation because the encounters were approximately normally distributed around noon.

We ran two mixed-effects linear models to evaluate whether intergroup encounter outcomes were associated with C-peptide values. The dependent variable in the first model was urinary C-peptide, adjusted by specific gravity, and log-transformed on account of the span of values across four orders of magnitude (min and max: 0.11 and 176.45 ng sg^−1^). The fixed effects in this model were time (relative to the encounter and standardized to facilitate interpretation of the coefficients) and a categorical variable for encounter outcome (lose, win, draw, or control). We tested an interaction between these variables because we expected winning and losing groups to exhibit different patterns of change over time, but ultimately dropped the interaction because it was not significant. The crossed random effects in this model were encounter ID and group ID, which controlled for the consistent differences in C-peptide levels across groups and the fact that urine samples that were associated in time through an intergroup encounter were much more similar to each other than to samples from the same group at a different point in time.

To evaluate whether differences in energetic condition, relative to monthly averages, correspond to the outcomes of intergroup encounters, we evaluate the percentage difference between C-peptide levels and the mean value per group-period. This approach was necessary, in part, because of the dramatic variation in C-peptide levels across seasons and groups. We then used this measure of relative C-peptide as the dependent variable in the second model, which contained time (relative to the encounter), outcome and the interaction of these two variables as the fixed effects. The only random effect in this model was encounter ID. The second model allowed us to investigate relatively short-term changes in C-peptide values, whereas the first model examines long-term patterns of variation. We did not include a random effect for individual ID or a fixed effect for age or sex class, because these factors do not explain variation in C-peptide levels (M Brown, R Steinitz 2021, unpublished analyses).

To determine whether travel distance varied as a function of encounter outcome, we calculated the total travel distance (i.e. the sum of all 30 min steps) on the day after each encounter. As with C-peptide values, mean daily travel distance varies dramatically across months, from 506 to 1256 m (mean ± s.d. = 890 ± 181 m on *N* = 679 group-days with at least 10 h of location data), likely as a function of food availability. To control for this variation, we calculated the mean travel distance per group, per observation period, and then determined the percent difference between the post-encounter travel distance and the group-period mean. No such correction was necessary to compare pre-encounter travel distance across encounters that were won, lost or that ended in a draw: groups cannot travel very far in the 30 min before the start of the encounter so there is limited variation in this parameter, and there is no *a priori* reason to expect it to vary across seasons. Although the ideal method would have been to calculate the amount of time each group spent in the contest location prior to the encounter, this was often not possible if groups met early in the day or if we had difficulty locating the group in the morning. Given the limited sample size of pre-encounter travel distances, we used *t*-tests to compare travel distances as a function of encounter outcome. We used a one-tailed *t*-test to compare post-encounter travel for winning and losing groups. The summary data on travel distance suggested a surprising pattern, so we ran two-tailed *post hoc t*-tests to compare travel distance against a hypothesized difference-from-the-mean of 0. We conducted all statistical tests in STATA v. 17 (StatCorp LLC, College Station, TX) with *α* = 0.05.

## 3. Results

We observed 83 complete encounters among red-tailed monkey groups, with data on both groups in 14 of these encounters, for a total of 97 observations. Of the 83 encounters, approximately half ended in a win/loss outcome (displacement: *N* = 38 [46% of encounters]; deflection: *N* = 6 [7%]; mutual ignore: *N* = 38 [46%]; mutual avoid: *N* = 1 [1%]). For 62 of the encounters, we collected urine samples from at least one group on the same day (*N* = 136 samples) or the following day (*N* = 101 samples).

**Figure 1.**
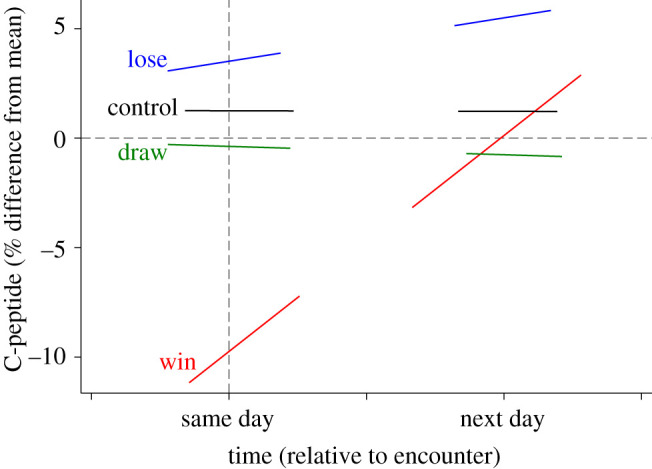
Model-fitted predictions for changes in relative C-peptide values when groups experience intergroup conflicts with win, loss, or draw outcomes, compared to control days without encounters. The dashed vertical line indicates the start time of the intergroup encounter. The break between samples collected on the ‘same day’ and the ‘next day’ is a product of the night-time gap in observations.

The ‘winners benefit’ perspective predicts that winning groups experience higher C-peptide levels after an encounter than losing groups (P1a), but we did not find support for this pattern (table 1; electronic supplementary material, table S2). The absolute values of C-peptide did not differ between winning and losing groups, which fits with the ‘everybody benefits’ prediction (P2a). The relative values indicated that on the day of the encounter—both before and immediately after—winning groups had lower-than-average values whereas losing groups had higher-than-average values (table 1 and figure 1; electronic supplementary material, table S2). The relative C-peptide values for winning groups improved dramatically after the encounter and reached average levels 24 h later (as predicted, P1b). The ‘winners benefit’ perspective also predicts that losing groups experience a decline in their pre- to post-encounter C-peptide levels (P1c), but we find no evidence of such a pattern: neither their absolute nor relative C-peptide values changed over time. These models indicate no change in C-peptide levels for draw outcomes, in contrast with prediction P3a.

**Table 1.** Models evaluating the relationship between intergroup encounter outcomes and urinary C-peptide values. Fixed effects are shown as coefficients and 95% confidence intervals (in parentheses). Predictors with coefficients and confidence intervals not crossing zero are indicated in bold and italic font.

response variable	absolute C-peptide	relative C-peptide
*N*	523	523
Wald *χ*^2^	0.85	31.19
d.f.	8	10
*fixed effects*		
time (relative to encounter)	0.06 (−0.28, 0.41)	3.15 (−5.87, 12.16)
loss versus win	0.06 (−0.55, 0.67)	***−13.39*** (−***19.47, −7.31***)
loss versus draw	−0.12 (−0.86, 0.61)	−3.85 (−10.47, 2.78)
loss versus control	−0.18 (−0.83, 0.46)	−2.23 (−8.02, 3.57)
time × win	—	***12.51*** (***0.08, 24.93***)
time × draw	—	−3.75 (−15.76, 8.26)
time × control	—	−3.19 (−12.89, 6.52)
intercept	***0.92*** (***0.13, 1.71***)	3.48 (−1.56, 8.52)
*random effects*		
intergroup encounter ID	***0.92*** (***0.75, 1. 12***)	***7.47*** (***6.10, 9.14***)
group ID	***0.67*** (***0.33, 1.34***)	—
residual	***1.15*** (***1.07, 1.23***)	***9.59*** (***8.97, 10.25***)

The ‘winners benefit’ perspective predicts that losing groups travel further the next day (P1d) to compensate for the energy shortfall associated with the intergroup encounter, but this was not the pattern that we observed (one-tailed *t*-test: *t* = −1.91, d.f. = 32, *p* = 0.97). A *post hoc* comparison against the focal group's mean travel distance in that observation period shows that the distance travelled by winning groups was no different from the mean (table 2; two-tailed *t*-test against the hypothesized mean: *t* = 0.99, d.f. = 21, *p* = 0.33), whereas losing groups travelled less than the mean distance (*t* = −2.45, d.f. = 11, *p* = 0.03), which also contradicts the ‘everybody benefits’ prediction (P2c). There was no change in travel distance on the day after an encounter ending in a draw (contradicting P3b; *t* = −1.37, d.f. = 29, *p* = 0.18).

**Table 2.** Travel patterns associated with win, loss and draw outcomes for intergroup encounters. Pre-encounter travel refers to the 30 min period before the start of an encounter. Post-encounter travel refers to the total distance travelled in 11 h on the day following the encounter, scaled as the percent difference from the mean travel distance for the focal group in the same month. Numbers within each cell are the mean ± s.e.m., followed by the sample size in parentheses.

	win	loss	draw
pre-encounter travel	67.5 ± 11.4 m (29)	36.0 ± 11.9 m (21)	48.4 ± 6.1 m (46)
post-encounter travel	+5.6 ± 5.7% (22)	−10.3 ± 4.2% (12)	−9.1 ± 6.6% (30)

The ‘everybody benefits’ perspective predicts that winning groups travel further than losing groups before encounters (P2b). This was indeed the case (table 2; one-tailed *t*-test: *t* = −1.88, d.f. = 48, *p* = 0.03).

## 4. Discussion

In contrast with widely held assumptions about the negative consequences of losing an intergroup contest over food resources, red-tailed monkey groups that lost an encounter did not experience reduced energy balance or increased travel effort. In fact, their energy balance was higher than average and their post-encounter travel distance was less than average, which indicates that they did not need to travel as far to meet their energetic needs. Winners started off with lower-than-average energy balance but experienced dramatic increases afterwards, reaching average levels 24 h later. Groups won if they had recently arrived at the feeding site, and lost if they had been in the site for at least 30 min (and presumably had an opportunity to feed on local resources). This pattern of results partially supports both the ‘winners benefit’ and ‘everybody benefits’ perspectives on the nature of contested food resources: winners reap energetic benefits, but losers do not experience energetic costs. In short, groups in poor energetic condition win whereas groups in better condition lose.

The effect of pre-encounter movement that we found here echoes a previous observation from the same forest: initiation of intergroup conflicts by grey-cheeked mangabeys depended upon whether they had recently arrived at the encounter location [44]. Red-tailed monkeys and grey-cheeked mangabeys eat many of the same foods [17], so the fact that immediate residency predicts the start and end of contests is striking. We interpret these patterns to mean that food trees cannot be depleted by a single group [24,45]. Our results also support the interpretation that large food trees cannot be monopolized continuously, because the winning group often supplants the losing group at the resource.

If both winning and losing groups have an opportunity to consume local resources, why do they waste energy on fighting instead of simply co-feeding or waiting until the departure of the other group? The answer may lie in the potential costs of these alternative strategies. By waiting, the newly arrived (and perhaps hungry) group loses potential feeding time. Co-feeding would mean more bodies in the tree simultaneously, which would increase the potential for aggressive interactions over food [46] or access to reproductive partners [47] while also making it harder for an individual to find and move to the ripest fruits in the crown [48]. Moreover, travelling to an alternative food source may be a poor option, particularly if other feeding sites are distant [19]. Evicting the resident group may be the best option, allowing the winning group to improve its energy balance rather than suffer additional deficits associated with non-confrontational alternatives.

By using a very short-term proxy for energetic condition, we isolated the important difference in the immediate, perceived resource value to winners and losers. For other species, long-term measures of motivation predict contest outcomes: more centrally located contest sites elicit greater effort from the resident group, leading to a higher probability of winning the encounter [13,14,18,33]. In contrast, red-tailed monkeys are no more aggressive in central home range areas than in peripheral areas, indicating that the long-term value of a resource may be less important than its immediate value [17]. It is unclear why red-tailed monkeys exhibit this pattern, and whether other species are as strongly influenced by the immediate value of the resource. More generally, while the ability to predict which group will win a contest may be useful, it is more important to measure the costs and benefits associated with outcomes in order to better understand the biological significance of intergroup contests [4,8,49]. Relative to many other group-living animals, primates are long-lived with slow reproductive rates, which makes it challenging to evaluate the cumulative fitness effects of intergroup contests. For instance, analysis of the costs and benefits of chimpanzee encounters relied on 18 y [50] and 20 y [51] datasets. The advantage of C-peptide is that it provides a more immediate indication of the potential energetic ramifications of these contests.

Most analyses that attempt to predict the outcome of intergroup conflicts use some form of group size as an estimate of resource-holding potential [3,4,6,13,14,18,33,49]. Importantly, size alone does not predict outcomes, which are instead shaped by the interaction of payoff asymmetries with resource-holding potential asymmetries [13,18]. However, our results indicate that groups in temporarily poor energetic condition (i.e. with potentially lower resource-holding potential or greater motivation) are successful in conflicts; if there is also a tendency for individuals in large groups to be in poorer condition than those in small groups, the purported role of group size may reflect need rather than strength. There tend to be higher levels of scramble competition in larger groups [23], along with higher rates of defection during intergroup conflicts [52,53], so there might also be greater variance in energetic condition among individuals. Thus there is a need for closer examination of short-term motivation as a function of group size, and to understand how individual condition affects group-level behaviours.

In a related analysis of red-tailed monkey intergroup conflicts, individuals experienced elevated cortisol levels after conflicts, regardless of whether their group won or lost [36]. However, cortisol is sensitive to both metabolic and psychological stressors so it was unclear whether red-tailed monkeys experience intergroup conflicts as energetically costly. Guenons (such as red-tailed monkeys) exhibit an intensity of agitation and hostility during aggressive between-group conflicts that is almost never seen during within-group interactions. They also engage in grooming frenzies after an intergroup conflict [54], presumably as a stress-relieving coping strategy [55], which further implies a strong psychological cost to these encounters. The fact that C-peptide does not decline for winning or losing groups indicates that the energetic cost of these encounters is likely to be minimal. Instead, winning groups experience energetic benefits that compensate for earlier deficits, and losing groups suffer no extraordinary costs. The short-term energetic causes and consequences of intergroup conflicts are clear, and support the interpretation that these conflicts are psychologically stressful but worthwhile for the potential energetic payoffs.

## Data Availability

The data and analytical code are available from the Dryad Digital Repository https://doi.org/10.25349/D91891 [56].
